# Supplementary material for: Effect of Treatment Methods on Material Properties and Performance of Sawdust-Concrete and Sawdust-Polymer Composites
Source: Polymers (Basel). 2024 Nov 26;16(23):3289. doi: 10.3390/polym16233289 (PMC11644647; doi:10.3390/polym16233289)
Supplement: Supplementary file 1 [file polymers-16-03289-s001.zip › polymers-3295543-supplementary.pdf]

Supplementary File

Table S1. Mechanical and physical properties of chemically treated SDC and SDPC

`	Description	Reference
NaOH	NaOH treated sawdust and untreated blast furnace were used to replace the fine aggregate and portland cement respectively with different composition.Deterrmined the best combination based on mechanical and physical characteristics.	[1]
	Sawdust-Polyster resin composites made from three different types plant where each type of sawdust treated with 10%NaOH solution.	[2]
	Impact of Filler material addition in polymer epoxy composites has been studied based on tensile performance where 5% NaOH treated Mahagoni sawdust and oil bean pod shell acted as filler.	[3]
	Compared the NaOH treated sawdust -high density polypropylene composites with composites derived from untretaed sawdust.Again, investigated the best applicable NaOH solution form 1,3,5,7 wt.% composition result.	[4]
	NaoH with boiling water was used to treat the sawdust and choose the best combination from different NaOH concentration and boiling time based on sawdust-paper waste hybrid composites mechanical properties.	[5]
	Analysis the effect of sawdust addition in sawdust-Acrylonitrile Styrene(ABS) polymer composties properties where sawdust treated with 5wt.% NaOH solution.	[6]
	Different NaOH concentration were considered for Sawdust treatment in sawdust-polyster composites.Again, Comparative study between treated sawdust composites and untreated sawdust composites was made depending their exhibited results.	[7]
	10% NaOH chemical treatment on sawdust and analysized the effect of treatment on Sawdust-oil bean pod Shell(OBPD) composite's mechanical properties.	[8]
	Sawdust treated with 5% NaOH solution and the influence of using Flame retardant agent with treated sawdust was investigated considering their combustion process,thermal stability and mechanical charateristics.	[9]

	5% NaOH Treated sawdust composite exhibited result compared with untreated composites.Nevertheless, effect of fiber addition in sawdust-Prolypopylene(pp) was determined.	[10]
	Investigate the fiber volume addition impact on sawdust-polypropylene(PP) composite's properties wher saw dust treated with 5%NaOH solution.	[11]
NaOH+KOH+ H2O2	5% NaOH,20% H2O2 and 3.5 g/L KOH were used sequentially to treat the sawdust. Feasibility check of using different weighted sawsdut content in redmud-sawdust composties carried out with thermal,mechanical and morphological observations.	[12]
NaOH+C14H10O4 (benzoyl peroxide)	Effect of 5 Wt.% NaOH treated Teak wood sawdust in sawdust-high density Prolypopylene(HDPE) composites mechanical and physical properties.	[13]
NaOH+ Dimethyldichlorosilane (DMDCS)+ Polydimethylsiloxane (PDMS)	Determined the varying concentration and treatment time of PDMS and DMDCS on chinese fair wood block by comparing the mechanical properties and morphological observations where NaOH remains fixed in all combinations.	[14]
Ca(OH)2	Seven different composition of Ca(OH)2 treated sawdust were used to replace the cement in concrete mixture and result observed for 7,14,28 days individually.	[15]
	20% Ca(OH)2 boiled with water considered for sawdust treatment in sawdust concrete. A comparitive study between Ca(OH)2 treated concrete and water proofing agent diluted in kerosen treated concrete based on mechanical and physical performance.	[16]

Ca(OH)2,CaCl2,distilled water	Three different types of sawdust concrete mixture were formed treated with Ca(OH)2,CaCl2,distilled water individually and made comparison with concrol concrete mixture (without any sawdust) in terms of acquired mechanical properties.	[17]
Sodium Silicate	Natural sand replaced by treated sawdust with different composition and determined the best combination based on mechanical performance.For chemical treatment Sodium silicate and water used individually and a comparative study has been done.	[18]
Ca(OH)2+Sodium Silicate	Three different types of wood derived sawdust undergo a chemical treatment including Ca(OH)2+ 1% sodium silicate+10%sodium silicate mixture to make a cement paste with portland cement and effect of fiber addition was analyzed.	[19]
NaOCl	15% NaOCl treatment with and without water bath analyzed on sawdust concrete performance again effect of treated fiber addition was investigated.	[20]
Maleic Anhydride (C4H2O3)	Maleic Anhydride treatment on Ayous sawdust-Carbon nanotube hybrid composites has been studied through morphological analysis.	[21]
HCl+NH4OH+polyethylenimine(PEI)	Sawdust treated with HCl, NH4OH,Polyethylenimin solution individually to investigate the best treated sawdust in Polypropylene-sawdust composites.	[22]
Ethanol+Nacl +Sulfuric Acid	Three different types of wood derived sawdust undergo a chemical treatment stepwise with Ethanol solution,NaCl solution and Sulfuric acid solution to find out best feasible sawdust which compatibe with cement based on good interfacial adhesion and physical characteristics.	[23]
Ethanol+Tolune+H2O2+ Acetic Acid	Comparison between raw sawdust polyster composites and delignified sawdust composites in terms of mechanical performance.Again effect of fiber loading in composites was analysed.	[24]

Potassium methyl siliconate(PMS)	Investigate the impact of using PMS treated sawdust with plastic composites by determining morphological structure and compability analysis.	[25]
Detersive Solvent	A comparative study between detergent treated sawdust-jute composites and untreated composites was carried out as well as mechanical and microscopical analysis has been done.	[26]
CaCO3+Vinyltriethoxysilane (VTES), and diethyl ether	Effectiveness of using VETS+Diethyl ether treatment on rubber wood sawdust determined by comaparing with raw sawdust-HDPE hybrid composites interfacial bonding and tensile strength.	[27]
H2O2+Acetic Acid+KOH+ TiO2+Acrilonitrile	Cyanoethyl cellulose fibers oiented unsaturated polyster composites andwood pulp unsaturated polyster composoites mechanical performance and thermal stability was comapred.	[28]

References:

1. Shanmuga Priya, D.; Sakthieswaran, N.; Ganesh Babu, O. Experimental Study on Mortar as Partial Replacement Using Sawdust Powder and GGBS. *Mater Today Proc* 2021, *37*, 1051–1055, doi:10.1016/j.matpr.2020.06.292.

2. Hossain, M.F.; Islam, M.K.; Islam, M.A. Effect of Chemical Treatment on the Mechanical and Physical Properties of Wood Saw Dust Particles Reinforced Polymer Matrix Composites. *Procedia Eng* 2014, *90*, 39–45, doi:10.1016/j.proeng.2014.11.811.

3. Benjamin, U. Tensile Behaviour of Oil Bean Pod Shell and Mahogany Sawdust Reinforced Epoxy Resin Composite. *International Journal of Science, Technology and Society* 2019, *7*, 1, doi:10.11648/j.ijsts.20190701.11.

4. Jaya, H.; OMAR, M.F.; Md Akil, H.; Ahmad, Z.A.; Zulkepli, N.N. Effect of Alkaline Treatment on Sawdust Reinforced High Density Polyethylene Composite under Wide Strain Rate. *Materials Science Forum* 2016, *840*, 103–107, doi:10.4028/www.scientific.net/MSF.840.103.

5. Aigbomian, E.P.; Fan, M. Development of Wood-Crete from Treated Sawdust. *Constr Build Mater* 2014, *52*, 353–360, doi:10.1016/j.conbuildmat.2013.11.025.
6. Neher, B.; Nova, N.T.; Hossain, R.; Gafur, M.A.; Ahmed, F. Fabrication and Characterization on Physico-Mechanical and Structural Properties of Sawdust Reinforced Acrylonitrile Butadiene Styrene (ABS) Composites. *Materials Sciences and Applications* 2020, *11*, 644–658, doi:10.4236/msa.2020.119043.
7. Haque, M.E.; Khan, M.W.; Kabir Chowdhury, M.N. Synthesis, Characterization, Biocompatibility, Thermal and Mechanical Performances of Sawdust Reinforced Composite. *Polym Test* 2020, *91*, 106764, doi:10.1016/j.polymertesting.2020.106764.
8. Oghenerukevwe Prosper, E.; Uguru, H. Effect of Fillers Loading on the Mechanical Properties of Hardwood Sawdust/Oil Bean Shell Reinforced Epoxy Hybrid Composites. 2018, *8*, 620–626.
9. Shulga, G.; Neiberte, B.; Jaunslavietis, J.; Verovkins, A.; Vitolina, S.; Shakels, V.; Livcha, S. *Lignin-Containing Adhesion Enhancer for Wood-Plastic Composites*;
10. Rezaur Rahman, Md.; Nazrul Islam, Md.; Monimul Huque, Md. Influence of Fiber Treatment on the Mechanical and Morphological Properties of Sawdust Reinforced Polypropylene Composites. *J Polym Environ* 2010, *18*, 443–450, doi:10.1007/s10924-010-0230-z.
11. Ferede, E. Evaluation of Mechanical and Water Absorption Properties of Alkaline-Treated Sawdust-Reinforced Polypropylene Composite. *Journal of Engineering* 2020, *2020*, 1–8, doi:10.1155/2020/3706176.
12. Kong, I.; Khoo, K.M.; Buddrick, O.; Baharuddin, A.A.; Khalili, P. Synthesis and Characterization of Red Mud and Sawdust Based Geopolymer Composites as Potential Construction Material. *Materials Science Forum* 2018, *923*, 130–134, doi:10.4028/www.scientific.net/MSF.923.130.
13. Akter, T.; Nur, H.P.; Sultana, S.; Islam, Md.R.; Abedin, Md.J.; Islam, Z. Evaluation of Mechanical Properties of Both Benzoyl Peroxide Treated and Untreated Teak Sawdust Reinforced High Density Polyethylene Composites. *Cellulose* 2018, *25*, 1171–1184, doi:10.1007/s10570-017-1620-3.
14. Yang, R.; Zhang, J.; Wang, S.; Mao, H.; Shi, Y.; Zhou, D. *Hydrophobic Chinese Fir Wood*; 2018; Vol. 13;.
15. Mahmoud, S.Y.M.; Alshiekh, E.T.A.M. Eco-Friendly Concrete Using Local Materials From Sudan. In Proceedings of the Proceedings of the International Conference on Civil Infrastructure and Construction (CIC 2020); Qatar University Press, February 2020; pp. 900–908.
16. Saeed, H.H. *Pretreatment of Sawdust for Producing Sawdust Concrete*; 2013; Vol. 31;.
17. Hewayde, E.; Kubba, Z. Mechanical Properties of Concrete Incorporating Pre-Treated Wastes Sawdust. *Key Eng Mater* 2021, *895*, 147–156, doi:10.4028/www.scientific.net/KEM.895.147.
18. Siddique, R.; Singh, M.; Mehta, S.; Belarbi, R. Utilization of Treated Saw Dust in Concrete as Partial Replacement of Natural Sand. *J Clean Prod* 2020, *261*, 121226, doi:10.1016/j.jclepro.2020.121226.

19. Dias, S.; Almeida, J.; Santos, B.; Humbert, P.; Tadeu, A.; António, J.; de Brito, J.; Pinhão, P. Lightweight Cement Composites Containing End-of-Life Treated Wood – Leaching, Hydration and Mechanical Tests. *Constr Build Mater* 2022, *317*, 125931, doi:10.1016/j.conbuildmat.2021.125931.

20. Hassen, S.A.; Hameed, S.A. Physical and Mechanical Properties of Sawdust Cement Mortar Treated with Hypochlorite. *IOP Conf Ser Mater Sci Eng* 2020, *745*, 012149, doi:10.1088/1757-899X/745/1/012149.

21. Fozing Mekeuo, G.A.; Despas, C.; Péguy Nanseu-Njiki, C.; Walcarius, A.; Ngameni, E. Preparation of Functionalized *Ayous* Sawdust-carbon Nanotubes Composite for the Electrochemical Determination of Carbendazim Pesticide. *Electroanalysis* 2022, *34*, 667–676, doi:10.1002/elan.202100262.

22. Rahman, Md.R.; Ting, J.S.H.; Hamdan, S.; Hasan, M.; Salleh, S.F.; Rahman, Md.M. Impact of Delignification on Mechanical, Morphological, and Thermal Properties of Wood Sawdust Reinforced Unsaturated Polyester Composites. *Journal of Vinyl and Additive Technology* 2018, *24*, 185–191, doi:10.1002/vnl.21545.

23. Antwi-Boasiako, C.; Ofosuhene, L.; Boadu, K.B. Suitability of Sawdust from Three Tropical Timbers for Wood-Cement Composites. *Journal of Sustainable Forestry* 2018, *37*, 414–428, doi:10.1080/10549811.2018.1427112.

24. Rahman, M.R.; Hamdan, S.; Ngaini, Z.B.; Jayamani, E.; Kakar, A.; Bakri, M.K. Bin; Yusof, F.A.B.M. Cellulose Fiber-Reinforced Thermosetting Composites: Impact of Cyanoethyl Modification on Mechanical, Thermal and Morphological Properties. *Polymer Bulletin* 2019, *76*, 4295–4311, doi:10.1007/s00289-018-2598-1.

25. Piao, C.; Cai, Z.; Stark, N.M.; Monlezun, C.J. Dimensional Stability of Wood-Plastic Composites Reinforced with Potassium Methyl Siliconate Modified Fiber and Sawdust Made from Beetle-Killed Trees. *European Journal of Wood and Wood Products* 2014, *72*, 165–176, doi:10.1007/s00107-013-0736-x.

26. Ganesan, V.; Shanmugam, V.; Kaliyamoorthy, B.; Sanjeevi, S.; Shanmugam, S.K.; Alagumalai, V.; Krishnamoorthy, Y.; Försth, M.; Sas, G.; Javad Razavi, S.M.; et al. Optimisation of Mechanical Properties in Saw-Dust/Woven-Jute Fibre/Polyester Structural Composites under Liquid Nitrogen Environment Using Response Surface Methodology. *Polymers (Basel)* 2021, *13*, 2471, doi:10.3390/polym13152471.

27. Kusuktham, B. Mechanical Properties and Morphologies of High Density Polyethylene Reinforced with Calcium Carbonate and Sawdust Compatibilized with Vinyltriethoxysilane. *Silicon* 2019, *11*, 1997–2013, doi:10.1007/s12633-018-0020-0.

28. Rezaur Rahman, Md.; Nazrul Islam, Md.; Monimul Huque, Md. Influence of Fiber Treatment on the Mechanical and Morphological Properties of Sawdust Reinforced Polypropylene Composites. *J Polym Environ* 2010, *18*, 443–450, doi:10.1007/s10924-010-0230-z.
